# Mental Manifestations and Biomarkers of Alcohol Consumption

**DOI:** 10.3390/life14070873

**Published:** 2024-07-12

**Authors:** Ana-Maria Sarpe (Paduraru), Cristina Dodul, Emil-Andrei Vlase, Cristian Onișor, Elena Niculet, Octavian Catalin Ciobotaru, Eduard Polea Drima

**Affiliations:** 1 Faculty of Medicine and Pharmacy, “Dunarea de Jos” University of Galati, 35 Al. I. Cuza Street, 800008 Galati, Romania; cd302@student.ugal.ro (C.D.); cristian.onisor@ugal.ro (C.O.); elena.niculet@ugal.ro (E.N.); octavian.ciobotaru@ugal.ro (O.C.C.); 2 “Sf. Apostol Andrei” Emergency County Clinical Hospital Galati, 177 Brailei Street, 800578 Galati, Romania; emilandrei.vlase@gmail.com; 3 Railway General Hospital–Galati, 5-7 Alexandru Moruzzi Street, 800223 Galati, Romania; 4 “Elisabeta Doamna” Clinical Hospital of Psychiatry Galati, 290 Traian Street, 800179 Galati, Romania; eduard.drima@ugal.ro

**Keywords:** liver disease, biomarkers, alcohol withdrawal

## Abstract

The purpose of this study is to examine healthcare usage, morbidities, and alcohol consumption monitoring in patients before a diagnosis of mental manifestations to assist in the early identification of individuals at risk. Biological markers of alcoholism are separated into two groups: those biological variables that highlight with great confidence and validate the presence of a predisposition to alcoholism, also called trait markers, or those that highlight alcohol consumption, called markers of alcoholism (“status markers”). Biomarkers are the true “gold standard” for the diagnosis of alcoholism. They are valuable for tracking evolution and progress during biological and psychological therapy and for highlighting relapse. This review compiles the existing data from research on healthcare utilization, comorbidities, and alcohol consumption monitoring in patients before a diagnosis of mental manifestations to aid in the early identification of individuals at risk. This documentary study took place over three to four months by searching for terms on the Science Direct platform, PubMed, Web of Science, and Google Scholar such as alcoholism, alcohol use disorders, alcohol abuse, and biomarkers. Studies reporting on the development, characteristics, and utilization of blood biomarkers for alcohol consumption were included in the search. The initial search included a number of 11.019 articles that contained the keywords biomarkers and alcohol. Finally, a total of 50 research articles were considered. I am involved in clinical studies, meta-analyses, reviews, and case studies regarding alcohol consumption detection, as well as potential alcohol markers.

## 1. Introduction

**Figure 1:**
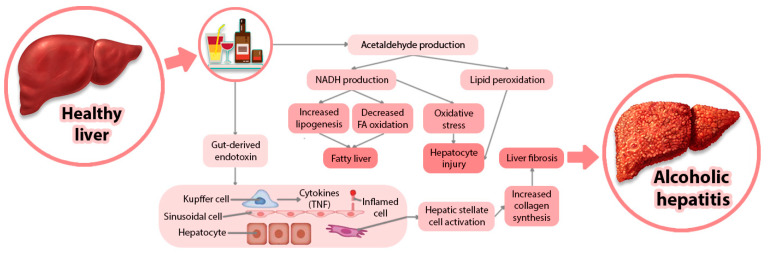
Pathogenesis of alcoholic liver disease.

Alcoholism has serious negative repercussions and difficulties, making it a global social and health concern [1]. The mechanism of action of alcohol on the liver has been studied in the specialty literature and is represented graphically in Figure 1.

Chemical laboratory analyses are a common procedure in clinical practice to accurately diagnose each patient. There are numerous signals for every analysis. Understanding the intricate link between environmental factors, disease, and clinical chemistry data is crucial given the scope of the analyses. One of the things that can cause variations in clinical chemistry values is alcohol usage [2].

Elevated levels of biomarkers are anticipated in chronic heavy alcohol users, as aspartate aminotransferase (AST), alanine aminotransfe (ALT), and gamma-glutamyltransferase (GGT) concentrations increase due to acute alcoholic hepatitis. This relationship with alcohol consumption is well established and occasionally serves as a signal to perform tests such as AST, ALT, and GGT [2,3]. Knowing which clinical chemical analytes have a strong correlation with alcohol consumption is crucial for clinicians. Excessive alcohol intake can be the cause of a deviant result from routine tests that cannot be attributed to more common medical disorders [2,3].

This relationship with alcohol consumption is well established and occasionally serves as a signal to perform tests such as AST, ALT, and GGT [2,3]. Knowing which clinical chemical analytes have a strong correlation with alcohol consumption is crucial for clinicians. Excessive alcohol intake can be the cause of a deviant result from routine tests that cannot be attributed to more common medical disorders [2,3]. A set of biomarkers indicating an individual’s alcohol consumption has been available to doctors for a long time. Many of them are certain liver enzymes, including carbohydrate-deficient transferrin (CDT), a protein that has recently attracted attention, as well as GGT, AST, and ALT [4].

Alcohol contains large amounts of carbohydrates, which are broken down by liver cells and other cells using another marker called N-acetyl-hexosaminidase (beta-Hex) and corpuscular volume (CVM), a biomarker of alcohol consumption that has also been used by clinicians to measure red blood cell volume [4]. If practitioners are aware of these indicators, the use of alcohol screening together with knowledge of the early signs of alcohol psychosis should facilitate earlier identification and more effective targeting of individuals at risk.

According to estimates from the World Health Organization, 140 million or more individuals worldwide struggle with alcoholism [5]. It stands to reason that much work has gone into the early identification of those patients who may go on to experience severe alcohol-induced cognitive impairment [5]. Despite alcoholic psychosis being one of the most prevalent conditions globally, there is a surprising lack of extensive research into the diagnostic process for this disorder. Surprisingly, there is not that much research into the process of diagnosing alcoholic psychosis. To be more specific, few published studies describe healthcare utilization prior to alcohol psychosis, and even fewer describe the conditions that patients present with before developing the disease.

This review compiles the existing data from research on healthcare utilization, comorbidities, and alcohol consumption monitoring in patients before a diagnosis of mental manifestations, intending to aid in the early identification of individuals at risk.

## 2. Methods

A literature review was conducted on publications in English discussing biomarkers of alcohol consumption. The documentary study took place over three to four months by searching on the Science Direct platform, PubMed, Web of Science, and Google Scholar for terms such as alcoholism, alcohol use disorders, alcohol abuse biomarkers, and cirrhosis. Studies reporting on the development, characteristics, and utilization of blood biomarkers for alcohol consumption were included in the search. The initial search included a number of 11.019 articles that contained the keywords biomarkers and alcohol. Finally, a total of 50 research articles were considered. I am involved in clinical studies, meta-analyses, reviews, and case studies regarding alcohol consumption detection, as well as potential alcohol markers.

Only the relevant articles that met the following criteria were selected: new biomarkers that could be introduced into clinical practice with much higher sensitivity and specificity in detecting alcohol consumption than the usual biomarkers, which are also elevated in other pathologies, and the psychological manifestations encountered in alcohol consumers. Studies containing biomarkers found in liver diseases of viral etiology were excluded.

## 3. Alcohol-Related Disorders and Health Complications

In a study conducted by Gro Askgaard and colleagues in 2018, regarding the incidence by sex, they reported the inclusion of 36,044 hospitalized patients who had their first hospital contact related to alcohol-related issues (24,727 men and 11,317 women). The initial diagnosis of alcohol problems included intoxication for 41–42% of both men and women, harmful use for 20–21%, and dependence for 37–39%. During the follow-up period, these patients had 301,525 subsequent hospital contacts related to alcohol problems, and 1966 were diagnosed with alcoholic liver cirrhosis. Among the men included, 13,924 (56%) had a second hospital contact related to alcohol problems during the follow-up period, 10,382 (42%) had a third, 7114 (29%) had a fifth, and 4147 (17%) had ten or more contacts, with similar percentages found for women.

Alcohol use disorders (AUDs) are divided by severity into intoxication, harmful use, and dependence. The most common screening tool to detect alcohol use disorders is the Alcohol Use Disorders Identification Test (AUDIT) [6]. In a UK study of primary care patients, 24% scored a positive AUDIT of 8, indicating hazardous drinking [7]. Among those with a positive AUDIT who agreed to further testing for liver disease, 22% scored 16 on AUDIT, which is indicative of dependence. Among hospitalized patients, 6–8% have high AUDIT scores, indicating dependence [8,9].

Most of the epidemiological literature to date has dealt with the level of drinking and the incidence or mortality of liver cirrhosis [10]. It followed the epidemiological tradition of early studies by Lelbach and others [11,12], who, based on studies among people with alcohol use disorders, posited a clear association between the volume of alcohol use and liver cirrhosis [13]. This association was corroborated in more rigorous studies [10,13,14]. However, it remains to be determined whether there is a threshold for alcohol-related liver damage or if any amount of alcohol increases the risk of liver cirrhosis, a topic discussed recently [15,16,17]. In fact, the most recent meta-analysis on this topic is over 10 years old and found some evidence for a protective association at low levels of alcohol intake in men [10]. Furthermore, several large-scale studies have been published since then [18,19,20].

## 4. Biomarkers

Biomarkers—a biomarker represents, according to the National Institutes of Health Biomarkers Definitions Working Group, a characteristic that is objectively measured and evaluated as an indicator of normal biological processes, pathogenic processes, or pharmacologic responses to a therapeutic or toxic intervention [21].

Liver enzymes (such as GGT, AST, and ALT)—labs measure liver enzyme activity in serum or plasma, where higher activity indicates liver damage. The key tissue for alcohol-induced toxic consequences is the liver, as it is the site of alcohol metabolism. When moderate to excessive drinking is combined with alcohol consumption over time, liver disease is usually associated with elevated liver enzyme levels [22,23]. For instance, serum liver enzyme activity may rise above baseline after consuming five standard drinks per week (about 70 g alcohol/week) for males or four standard drinks per week (roughly 60 g alcohol/week) for women for several weeks [22].

In specialized literature, a series of characteristics have been proposed to define the specificity of biomarkers, which can be found in Table 1.

Low-carbohydrate isoforms of transferrin (CDT, carbohydrate-deficient transferrin), specifically desialylated transferrin, are well known for their high sensitivity and specificity (82% and 97%, respectively) in diagnosing alcohol dependence [26,27]. Although desialylated transferrin is a relatively new marker for alcohol abuse, its diagnostic utility is growing [28,29]. The FDA (Food and Drug Administration) has approved it as a standalone test in the USA for identifying heavy drinking [30]. Ethanol and its metabolites can impair the enzymes responsible for modifying transferrin (increasing sialidase activity and decreasing sialyltransferase) and disrupt the function of liver cell receptors that eliminate desialylated transferrin [28]. CDT comprises transferrin with reduced sialic acid content (asialo-, monosialo-, and disialotransferrin) [31]. Consuming 50 to 80 g of alcohol daily for at least one week, or more than 60 g/day for 7–10 days, significantly raises CDT levels in the serum. After a short period of abstinence, even small amounts of alcohol can cause a significant increase in serum levels again [26,27]. Therefore, CDT is considered more sensitive than GGT in detecting relapse drinking (monitoring abstinence) and differentiating between alcoholic and non-alcoholic liver injuries. However, CDT shows low sensitivity (12–45%) in the general population, including women, young people, binge drinkers, and healthy individuals, even at high alcohol doses [26]. As a screening test in the general population, CDT is not more sensitive than GGT, so its sensitivity is related to the level of CDT and the total level of transferrin (% CDT; CDT/total transferrin ratio) [26]. CDT levels return to normal within a few weeks of abstinence, with a half-life of approximately 15 days [26]. Certain diseases can reduce the specificity of the CDT test. These include non-alcoholic liver diseases (primary biliary cirrhosis, chronic active inflammation, HCV, and hepatocellular carcinoma), senile dementia, depression, pregnancy, solvent poisoning, genetic glycoprotein deficiency syndrome, pancreatic and kidney transplantation, cystic fibrosis, insulin-related metabolic disorders, iron deficiency, galactosemia, and anal cancer [26,32]. A study found that salivary CDT is not applicable as an alcohol marker [33].

The non-oxidative metabolism of ethanol results in the production of fatty acid ethyl esters (FAEEs). These compounds are created through the conjugation of fatty acyl chains (such as palmitic acid, oleic acid, and stearic acid) with ethanol. Enzymes such as FAEE synthase, microsomal acyl-CoA, O-acyltransferase, carboxylesterase, lipoprotein lipase, cholesterol esterase, and triglyceride lipase catalyze this reaction. However, FAEEs can also form spontaneously [34].

Recent studies have shown that fatty acid ethyl esters (FAEEs) are sensitive and specific markers for distinguishing between social drinkers and heavy or alcohol-dependent drinkers [35]. FAEE levels have been detected in the blood 24 h after the last drink, whereas blood ethanol levels only remain elevated for 8 h [36]. Moreover, FAEE levels have been observed to increase for at least 99 h after ethanol consumption in heavy drinkers [37]. Additionally, serum concentrations of ethyl oleate are higher in chronic alcohol users compared to binge drinkers, allowing for the differentiation between occasional binge drinkers and alcohol-dependent individuals [38]. Recent studies have also found that FAEE can be detected in hair for several months [39,40].

A relatively new biomarker called phosphatidylethanol (PEth) can detect alcohol concentrations in the blood within the range of 2–4 weeks after consumption [41,42]. PEth is a type of abnormal phospholipid found in cell membranes that forms only in the presence of ethanol [41]. The amount of PEth in the blood increases with repeated alcohol consumption, making it useful for distinguishing between excessive and moderate alcohol intake.

In a recent review of available biomarkers for detecting acute and chronic alcohol use, indirect biomarkers such as carbohydrate-deficient transferrin and mean corpuscular volume were found to have lower sensitivity and specificity compared to direct biomarkers like PEth for identifying unhealthy alcohol consumption [43].

Recent studies have shown that PEth formation occurs both after multiple consumption episodes and after a single consumption of small amounts of alcohol, resulting in blood alcohol concentrations (BACs) of up to 1 g/kg [44,45]. Javors et al. demonstrated PEth formation after single doses of 0.25 g or 0.5 g of ethanol per kg of body weight, with peak PEth concentrations observed between 90 and 120 min after alcohol consumption [46]. The authors used a limit of 5 ng/mL, while a limit of 20 ng/mL in the USA has been agreed upon [47,48]. The same researchers showed proportional increases in PEth concentration after a single alcohol consumption of 0.4 g/kg and 0.8 g/kg. However, PEth concentrations were above the quantification limit in most participants even before alcohol consumption began, indicating that the required abstinent period was not sufficient [49].

With alcohol abstinence, PEth levels gradually decrease, but the elimination half-life time varies significantly between individuals [50]. As a biomarker for long-term alcohol consumption, the PEth test indicates high alcohol intake over the past few weeks up to about a month, depending on the initial level and individual elimination rate. However, even a single instance of heavy drinking can generate measurable, though lower, levels.

Keratin, previously known as cytokeratin, forms the primary subgroup of epithelial-specific intermediate filament proteins, performing various functions, including protecting hepatocytes from apoptosis and necrosis [51]. In adult hepatocytes, only K8 and K18 are present, and K18 is released during cell death.

Recently, several researchers have evaluated K18 as a diagnostic and prognostic marker for alcoholic hepatitis (AH) [52]. A small study on alcohol-associated cirrhosis (AC) and AH showed direct correlations between increased K18 levels and greater severity of liver disease, as well as between increased K18 levels and mortality [53]. Bissonnette and his colleagues analyzed several potential biomarkers for AH in patients undergoing liver biopsy to diagnose suspected AH [54]. Keratin 18 (M65) was found to be the most useful biomarker evaluated, with a positive predictive value of 91% at an upper cutoff of 2000 IU/L and a negative predictive value of 88% at a lower cutoff of 641 IU/L. In this study, of the patients who did not have AH on biopsy, 95% had AC without steatohepatitis [54].

The authors concluded that the measurement of serum K18 levels should be integrated into routine clinical practice as it helps to identify patients with severe alcoholic hepatitis.

β-Hexosaminidase (β-HEX) is a lysosomal exoglycosidase present in most cell types and plays a role in the catabolism of glycoproteins, proteoglycans, etc., by releasing N-acetylhexosamines from the non-reducing end of their glycoconjugate oligosaccharide chains [4,55]. Consuming large amounts of alcohol, i.e., >60 g per day for at least 10 consecutive days, causes significant changes in enzymatic activity in body fluids. One proposed mechanism for this change is lysosomal damage, which then leads to the leakage of the enzyme from lysosomes and cells into body fluids [4]. Diagnostic sensitivity levels for the increase in β-HEX B activity in serum and β-HEX in urine have been established at 69–94% and 81–85%, respectively. Additionally, in alcohol-dependent individuals, enzyme levels drop dramatically during periods of sobriety (7–10 days, T½ = 6.5 days) [2,55]. However, β-HEX levels in saliva, urine, or serum can also increase after the isolated ingestion of approximately 2 g/kg of alcohol (also known as “binge drinking”) [56]. Despite the relatively high specificity (84–98%), individuals with liver diseases (such as cirrhosis and cholestasis), thyrotoxicosis, diabetes, hypertension, pregnancy, myocardial or cerebral infarction, and those taking oral contraceptives may show false-positive results due to increased enzymatic activity [2,55]. The unique advantage of β-HEX as a potential marker for prolonged alcohol abuse is that it is a simple and inexpensive detection technique. Other lysosomal exoglycosidases are potential alcohol markers, for example, isoenzyme A, β-hexosaminidase (HEX A) as a marker of chronic alcohol consumption intensity, α-fucosidase (FUC) and α-mannosidase (MAN) as markers of alcohol dependence, and β-glucuronidase (GLU) as a marker of both conditions simultaneously [56].

## 5. Mental Manifestations

Alcoholic psychosis can be a mental and behavioral disorder that results from continued alcohol addiction [57]. Compared to schizophrenia, the central points of the syndrome include delusions; visualizations, most often in contemptuous and threatening voices; disordered temperament; hindered thinking; and inappropriate conduct [58].

In alcohol use disorder (AUD) patients, the cessation of alcohol utilization is habitually related to a few clinical side effects (tremors, nausea, uneasiness, a sleeping disorder, etc.) that constitute the alcohol withdrawal disorder (AWS) [59].

The severity of AWS is variable in patients with AUD, ranging from a mild clinical setting to extreme neurological complications such as seizures and incoherence tremens, possibly leading to death [60]. Scenes of ridiculousness tremens have a mortality rate of 1% to 5%. Risk variables for creating alcohol withdrawal incoherence incorporate concurrent intense restorative sickness, overwhelming alcohol consumption every day, a history of dizziness or withdrawal seizures, more experienced age, abnormal liver function, and more extreme withdrawal side effects at introduction [61]. Distinctive evidence of early release of the drink allows for the prevention and treatment of complications, taking into account extreme withdrawal.

According to Tsuang et al. [62], who studied a cohort of 643 patients, the prevalence of acute alcoholic hallucinosis is 7.5% of all hospitalized patients with alcohol dependence. Meanwhile, Soyka estimated that acute alcoholic hallucinosis has been detected in 0.4% of patients hospitalized for alcohol dependence [63]. In a population-based study, Perälä et al. [64] found a lifetime prevalence of acute alcoholic hallucinosis of 0.41%. In the subpopulation of individuals with alcohol dependence, the prevalence rate was 4.0%. The highest lifetime prevalence of acute alcoholic hallucinosis was found in the 45–54 year age group (1.8%). According to the literature, it has been found that a younger age of onset of alcohol dependence, low socioeconomic status, family burden of alcohol use disorders and other mental disorders, as well as repeated hospital admissions due to alcohol use disorders, are associated with a higher risk of acute alcoholic hallucinosis [65].

Similar to the information regarding affective disorders, studies also show a significant prevalence of anxiety symptoms among individuals who are dependent on alcohol [53,66]. Some clinicians and researchers have inferred from these data that anxiety disorders and alcohol use disorders may have a genetic connection. Others have suggested that many people with major anxiety disorders use alcohol as a way to alleviate their symptoms [67].

However, it is not clear from the existing literature whether the high rate of anxiety problems appearing only in the context of heavy drinking or withdrawal indicates the existence of lifelong anxiety disorders that will require long-term treatment. The majority of these situations may involve temporary anxiety symptoms observed only during intoxication and withdrawal.

In several studies, 80% of alcohol-dependent men admitted to experiencing repetitive panic attacks during alcohol withdrawal, and 50–67% of these men scored high on state anxiety measures, displaying symptoms resembling generalized anxiety disorder and social phobia [68].

Patients aged 65 and older are at increased risk for complications related to alcohol withdrawal syndrome (AWS) due to a higher prevalence of comorbidities, including cognitive impairment, a longer history of alcohol consumption, and greater sensitivity to AWS treatment. Older patients with less physiological reserve may become symptomatic earlier due to the brain’s limited ability to adapt to stressors such as illness, trauma, or surgery. However, these notions are not universally applicable, and a higher index of suspicion for AWS should be maintained. Symptoms may appear earlier (6–12 h) from the last drink and can present with a wide range of manifestations. When symptoms manifest, AWS severity scores may be higher, and their duration is likely longer than in younger individuals [69].

Men and women with alcohol use disorder (AUD) are reported to have more than four times the risk of completing suicide compared to the general population, even after accounting for sociodemographic differences and other psychiatric and somatic disorders [70]. Due to these high risks and the overall high prevalence of AUD, it is estimated to account for 20% of all disability-adjusted life years (DALYs) lost due to suicide [71]. Little is known about healthcare utilization patterns before suicide among individuals with AUD. Understanding these patterns may reveal crucial opportunities to prevent suicide in this high-risk patient population.

Several studies have investigated healthcare utilization before suicide in general populations or patient samples. Most of these studies found that a significant majority (80–90%) of individuals who died by suicide had a healthcare encounter in the previous year, and approximately 30–40% had a primary care encounter in the previous month [72,73,74,75,76,77]. Healthcare utilization also varies across different psychiatric disorders and patient subgroups [74,75]. A U.S. study on male military veterans with substance use disorders (either AUD or drug use disorders) reported that 94.6% and 55.6% had a healthcare encounter within the year or month before suicide, respectively [78].

## 6. Discussions

Alcoholic liver disease is a consequence of excessive alcohol consumption, and treatment requires special attention for both the liver disease and the underlying mental disorder.

Alcohol has become the leading cause of cirrhosis in most high-income countries, accounting for about 50% of all cirrhosis deaths worldwide [79].

The amount of alcohol consumed, measured in grams per day, is proportional to the risk of developing liver disease [10]. Although not all patients who consume alcohol initially develop liver cirrhosis, the identification of this pathology is very important as these patients are at increased risk of portal hypertension, and screening for hepatocarcinoma is necessary. Sometimes the diagnosis of cirrhosis can be late, being diagnosed at the time of decompensation. In alcoholic liver disease, the chances of late diagnosis are 12 times higher than in viral hepatitis [80].

Ascites is the most common complication of cirrhosis of the liver and represents the pathological accumulation of fluid in the peritoneal cavity. Bacterial infections are 5–6 times more common in patients with liver cirrhosis and are a major cause of disease decompensation [81].

Spontaneous bacterial peritonitis (SBP) is defined as ascitic fluid infection in the absence of a surgically treatable intra-abdominal source [81].

Hepatic encephalopathy is the cerebral sequelae of liver failure in cirrhosis and develops due to the free passage of neurotoxins developed in the intestine through the diseased or shunted liver, thereby reaching the central nervous system due to dysfunction of the blood–brain barrier. Cirrhosis of the liver is the main cause of digestive hemorrhage (HD) [81].

Pulmonary complications of liver cirrhosis are hepatic hydrothorax, hepato-pulmonary syndrome, and porto-pulmonary hypertension [81].

Hepatocarcinoma (HCC) is a relatively common complication of cirrhosis. It should be noted that any new hepatic nodule arising in the background of cirrhosis of the liver should be considered HCC until proven otherwise [81].

Paraclinical investigations such as elevated bilirubin, elevated transaminases, elevated international normalized ratio (INR), and thrombocytopenia can be useful in the diagnosis of cirrhosis [82]. Thrombocytopenia is the earliest indicator, so a count of less than 160,000 platelets represents increased accuracy in the diagnosis of cirrhosis [76]. However, alcohol-using patients may present with thrombocytopenia due to direct alcohol toxicity, including myelosuppression and accelerated platelet degradation [83].

The biomarkers of alcohol consumption should indicate the predisposition, risk, and likelihood of a patient developing alcoholism in the near or distant future. This type of marker indicates a trait of the individual, a genetic, biochemical, or behavioral characteristic that can be detected throughout the life of the subject and not only during the period of alcohol consumption, so this marker must be genetically transmitted and must not be a side effect of alcohol consumption or alcoholism [84].

Alcohol consumption markers are markers of the subject’s “state” and reflect changes in the body as a result of alcohol consumption. Studies show that only 25–29% of patients with somatic disorders admit to excessive alcohol consumption [85,86]. This percentage increases in psychiatric inpatients admitted for alcohol consumption [87].

Biomarkers are commonly used in medicine for the diagnosis of various diseases, whether it is cancer [88] or liver pathologies caused by alcohol consumption [89]. Traditional biomarkers that aim to detect excessive alcohol consumption have been used for many years, but many lack sensitivity and specificity [89], unlike the new biomarkers mentioned in this article, which are much more specific for alcohol consumption compared to the others currently available.

Two out of three “alcoholics” meet the criteria for another major psychiatric disorder at some point in their lives [89,90,91,92].

Alcoholism is defined as a primary, chronic, often progressive, and even fatal disease. The medical complications of alcoholism are multiple. They are represented by gastritis, pneumonia, liver failure, ulcers, pancreatitis, cardiomyopathy, Korsakoff’s psychosis, alcoholic dementia, and an increased risk of cancers of the tongue, larynx, liver, and stomach [10]. Alcoholic disease is associated with anxiety, depression, and somatization disorders. Long-term consumption may precede anxiety disorders or panic attacks. Another important and, at the same time, severe manifestation of alcoholism is delirium tremens, which is characterized by temporal–spatial disorientation, confusion, perceptual disturbances, delusions, hallucinations, psychomotor agitation, fever, and insomnia. Symptoms appear 2–3 days after the cessation of heavy alcohol consumption, with maximum intensity in 4–5 days. Alcohol-induced psychotic disorder is characterized by the appearance of marked auditory hallucinations for at least a week, which begins after the reduction or cessation of excessive alcohol consumption. The patient responds to these hallucinations with fear, anxiety, and agitation [93].

In conclusion, we can state that alcoholism is prevalent, alcohol-induced liver disease is a major health problem, and the recently studied biomarkers are a real “gold standard” for the diagnosis of alcoholism, as well as being valuable for monitoring evolution and progress during biological and psychological therapy and in highlighting rehabilitation.

Biomarkers that detect alcohol consumption or harmful use of alcohol offer the possibility of verifying information about alcohol consumption provided by a patient objectively.

## 7. Future Research Directions

Future research should investigate new blood biomarkers that could potentially offer better specificity and sensitivity in measuring various aspects of alcohol consumption, including excessive drinking, periods of abstinence, and moderate alcohol intake. This review does not include other potential biomarkers, such as cytokines, because they are primarily used in research settings and exhibit inconsistent sensitivity and specificity. Additionally, redox markers are not covered here as they are typically used for assessing liver injury rather than directly measuring alcohol consumption.

Identifying new biomarkers that provide quantitative information about alcohol consumption patterns can enhance clinicians’ ability to detect, treat, and monitor alcohol use disorders (AUD). Currently available blood biomarkers are more specific for alcohol-related organ damage (e.g., alcohol-associated liver disease) than for risky alcohol consumption itself, highlighting a critical area for further research.

Examining blood biomarkers in the context of other disorders in patients with AUD can help validate their use in diverse populations with comorbid conditions such as alcohol-associated liver disease, cardiovascular disease, and other alcohol-related disorders. Conversely, there is a need to develop and validate more specific biomarkers for alcohol consumption among light-to-moderate drinkers who have not developed other medical comorbidities. While the current blood biomarkers can assess recent alcohol consumption, more sensitive and specific biomarkers are needed to measure varying levels of alcohol consumption over different time intervals. Additionally, biomarkers that can accurately detect and quantify alcohol consumption in the presence or absence of comorbid diseases are necessary.

Therefore, it is imperative to investigate combinations of existing biomarkers and identify new blood biomarkers that are more sensitive, specific, readily accessible, and cost-effective. These biomarkers should be used in conjunction with self-reported assessments to better characterize alcohol consumption.

The future promises revolutionary advances that will re-explore how we approach diagnosis and patient care. Thus, in exploring future directions, we delve into the realms of biomarkers and novel diagnostics. Profound advances in biomarkers result from relentless investigation.

Future research aims to identify potential biomarkers that could help in the early identification of individuals at risk of developing alcohol-related mental health issues, as well as to establish a connection between biomarker levels and psychological manifestations and to prevent alcoholic liver diseases.

## 8. Conclusions

This article presents a new perspective on detecting harmful alcohol consumption, identifying secondary clinical and paraclinical changes as early as possible, and increasing the predictability of the evolution of associated conditions. Early detection can significantly contribute to the reduction of alcohol consumption, the occurrence of organic changes, and, why not, the prevention of behavioral and cognitive changes.

The primary challenge in diagnosing alcohol use and alcohol use disorders in the future will be translating expensive and advanced analytical techniques for identifying specific compounds in readily available tissues and fluids into affordable and simple diagnostic tools for routine medical practice. Additionally, larger population studies are needed to assess the effectiveness of these new potential markers of alcohol use.

## Figures and Tables

**Table 1:** The quality criteria or a marker of alcohol consumption [24,25].

CRITERIA	SIGNIFICANCE
Accuracy	or validity; the correlation between the cases distinguished of the marker and the truly positive cases
Precision	or confidence; the ability of the marker to identify the cases that are truly positive in a heterogeneous population
Sensitivity	the proportion of patients without alcoholism that come out positive
Specificity	the proportion of patients without alcoholism that come out positive
Stability	the marker must persist at least a few days after stopping drinking and return to normal after a reasonable period
Practicability	the ease of obtaining biological evidence from which to check the presence of the marker
Disponibility	the ease with which the identifying of the marker can be performed
Low cost	the cost of the materials, the method and techniques, and the lab
Transportability	the ease of implementing of the technique in different, appropriate places
Non-invasive	the sampling of the biological evidence in which you can detect the marker must be as least invasive as possible for the organism
Acceptability	the desire of the patients to use this technique, and the desire to submit themselves to this test

## Data Availability

No new data were created or analyzed in this study.
